# Anti-Inflammatory Activities and Liver Protection of Alisol F and 25-Anhydroalisol F through the Inhibition of MAPK, STAT3, and NF-κB Activation In Vitro and In Vivo

**DOI:** 10.3390/molecules22060951

**Published:** 2017-06-08

**Authors:** Xiaoxu Bi, Pu Wang, Qingjuan Ma, Li Han, Xingbo Wang, Yu Mu, Peipei Guan, Xiaodan Qu, Zhanyou Wang, Xueshi Huang

**Affiliations:** College of Life and Health Sciences, Northeastern University, Shenyang 110819, China; xiaoxu.bi@hotmail.com (X.B.); wangpu@mail.neu.edu.cn (P.W.); maqingjuan0319@163.com (Q.M.); wangxbneu@hotmail.com (X.W.); muyu@mail.neu.edu.cn (Y.M.); guanpp@mail.neu.edu.cn (P.G.); xiaodanqu@hotmail.com (X.Q.); wangzy@mail.neu.edu.cn (Z.W.)

**Keywords:** alisol F, 25-anhydroalisol F, RAW 264.7 macrophages, anti-inflammatory, LPS/d-gal-induced acute liver injured mice, NF-κB, MAPKs, STAT3 signaling pathways

## Abstract

Alisol F and 25-anhydroalisol F isolated from *Alisma orientale*, were proved to exhibit anti-inflammatory potential in our previous work. In the current study, the anti-inflammatory effects and action mechanisms of alisol F and 25-anhydroalisol F were investigated in vitro. Moreover, the pharmacological effects of alisol F in lipopolysaccharide (LPS)/d-galactosamine (d-gal)-induced acute liver-injured mice were evaluated. The results demonstrated that alisol F and 25-anhydroalisol F could suppress LPS-induced production of nitric oxide (NO), interleukin-6 (IL-6), tumor necrosis factor alpha (TNF-α), and interleukin-1β (IL-1β), as well as inhibit the mRNA and protein levels of inducible nitric oxide (iNOS) and cyclooxygenase-2 (COX-2). In addition, we investigated the role of alisol F and 25-anhydroalisol F in mediating mitogen-activated protein kinases (MAPKs), signal transducers, and activators of transcription 3 (STAT3) and nuclear factor κB (NF-κB) pathways involved in the inflammation process of LPS-stimulated RAW 264.7 cells. The phosphorylation of ERK, JNK, p38, and STAT3, and the NF-κB signaling pathway, were obviously suppressed in alisol F and 25-anhydroalisol F treated cells. Results obtained from in vitro experiments suggested alisol F obviously improved liver pathological injury by inhibiting the production of TNF-α, IL-1β, and IL-6, and significantly decreasing the serum alanine aminotransferase (ALT) and aspartate aminotransferase (AST) levels in LPS/d-gal-induced mice. Furthermore, the reduction of phosphorylation of ERK and JNK, as well as suppression of the NF-κB signaling pathway, were also observed in liver tissues of the alisol F-treated mice model. Alisol F and 25-anhydroalisol F may serve as potential leads for development of anti-inflammatory agents for acute liver failure treatment.

## 1. Introduction

Inflammation is a complex defense process caused by many factors, such as chemical stimulus, physical damage, and bacterial infections [1]. However, excessive inflammation often causes many chronic and acute diseases, including atherosclerosis, arthritis, acute hepatitis, and multiple sclerosis [2,3,4]. Among them, the acute hepatic failure always leads to systemic inflammation through the release of pro-inflammatory cytokines, such as tumor necrosis factor-α (TNF-α), interleukin (IL)-1β, and IL-6 [5,6,7], which is an intractable disease with high mortality rates.

Lipopolysaccharide (LPS)/d-galactosamine (d-gal) are usually used to establish the experimental animal models of acute hepatic failure [6,8]. LPS usually binds to the Toll-like receptor 4 (TLR4)/CD14 complex, which induces the expression of inflammatory cytokines [9]. Of note, the induction of these inflammatory cytokines is mediated by the signal pathways such as mitogen-activated protein kinase (MAPK), nuclear factor κB (NF-κB) and Janus kinase/signal transducers and activators of transcription (JAK/STAT) [10,11]. Apart from LPS, d-gal acted as the hepatotoxic or liver damaging agent for establishing animal models of liver failure [12].

*Alismatis rhizoma*, the dried rhizome of *Alisma orientale* (Sam.) Juz. has been widely used as a remedy for hyperlipidemia, hyperglycemia, and inflammatory diseases in China [13]. The ethanol extract of *A. orientale* had been proved to reduce the acute lung inflammation by suppressing the NF-κB [14]. Protostane triterpenes were the primary chemical constituents of *A. orientale* and exhibited a number of biological activities, such as antivirus, antitumor, hepato-protective, and anti-inflammatory activities [15,16,17,18,19]. However, there were few reports on anti-inflammatory mechanism of protostane triterpenes, except for inhibiting the production of nitric oxide (NO) in lipopolysaccharide (LPS)-stimulated macrophages [15,20]. Our prior work demonstrated that two protostane triterpenes, alisol F and 25-anhydroalisol F, isolated from *A. orientale* exhibited anti-inflammatory activities [21]. In current study, we further investigate the mechanism and the liver protection of these two compounds in vitro and in vivo, using LPS-stimulated RAW 264.7 cells and LPS/d-gal-induced liver injury in mice.

## 2. Results and Discussion

### 2.1. Effect of Alisol F and 25-Anhydroalisol F on Viability of LPS-Stimulated RAW 264.7 Cells

**Figure 1:**
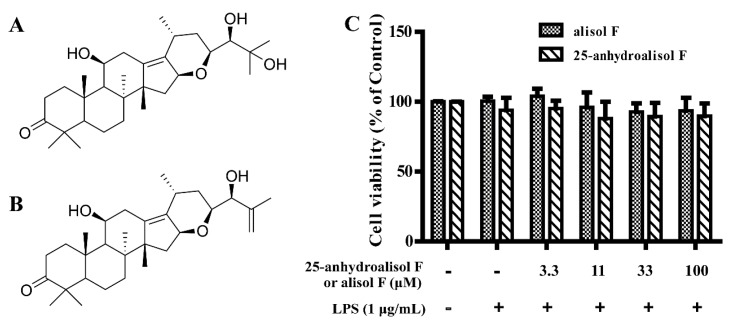
The effect of alisol F and 25-anhydroalisol F on cell viability of RAW 264.7 cells. The structures of alisol F and 25-anhydroalisol F are shown in (**A**,**B**); the cell viability was measured by MTT assay (**C**). Cells were pretreated with the different concentrations of alisol F (0, 3.3, 11, 33 and 100 μM) and 25-anhydroalisol F (0, 3.3, 11, 33 and 100 μM) for 2 h and then stimulated with or without LPS (1 μg/mL) for 24 h. The data represent the means ± S.E. values of three independent experiments.

Our prior work have demonstrated that alisol F and 25-anhydroalisol F had ability to inhibit the production of NO in LPS-stimulated RAW 264.7 cells [21]. To further evaluate the effects of alisol F and 25-anhydroalisol F on inflammation, the MTT assay was firstly carried out. The result showed that both of compounds had no cytotoxicity on RAW 264.7 cells within the range of 100 μM (Figure 1). Proliferating rates indicated that the anti-inflammatory effects of the two compounds were not due to the induction of apoptosis or cell death at the experimental dosages.

### 2.2. Alisol F and 25-Anhydroalisol F Inhibited the Production of Inflammatory Cytokines in LPS-Stimulated RAW 264.7 Cells

**Figure 2:**
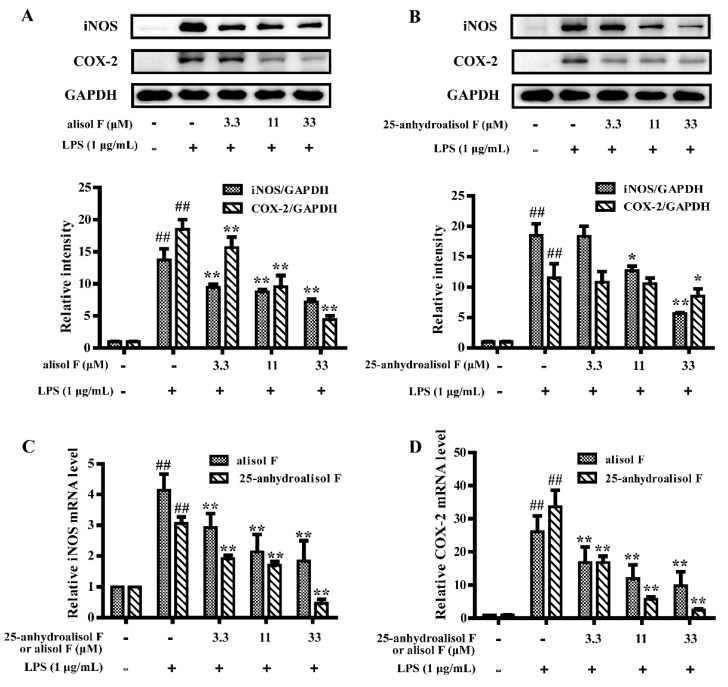
The effect of alisol F and 25-anhydroalisol F on the expression of iNOS and COX-2 in LPS-stimulated RAW 264.7 cells. Cells were pretreated with the various concentrations of alisol F (0, 3.3, 11 and 33 μM) and 25-anhydroalisol F (0, 3.3, 11 and 33 μM) for 2 h and then stimulated with or without LPS (1 μg/mL) for 24 h. The expression of iNOS and COX-2 protein levels were analyzed by Western blot (**A**,**B**); Cells were pretreated in the same way for 2 h and then stimulated with or without LPS (1 μg/mL) for 4 h. The expression of iNOS and COX-2 mRNA levels were measured by quantitative real-time PCR (**C**,**D**). The data represent the means ± S.E. of three independent experiments. (^##^ *p* < 0.001 vs. control group; * *p* < 0.05, ** *p* < 0.001 vs. LPS group).

Pro-inflammatory enzymes cyclooxygenase-2 (COX-2) and iNOS, which controlled production of prostanoids and NO, were involved in inflammatory events [22]. As the powerful roles of iNOS and COX-2 in inducing inflammation [5], experiments were carried out to determine the effects of alisol F and 25-anhydroalisol F on the mRNA and protein expression of iNOS and COX-2. As shown in Figure 2, LPS treatment markedly stimulated the expression of iNOS and COX-2 in RAW 264.7 cells. When we added alisol F and 25-anhydroalisol F to the cells, these two chemicals blocked the stimulatory effects of LPS on these two genes.

**Figure 3:**
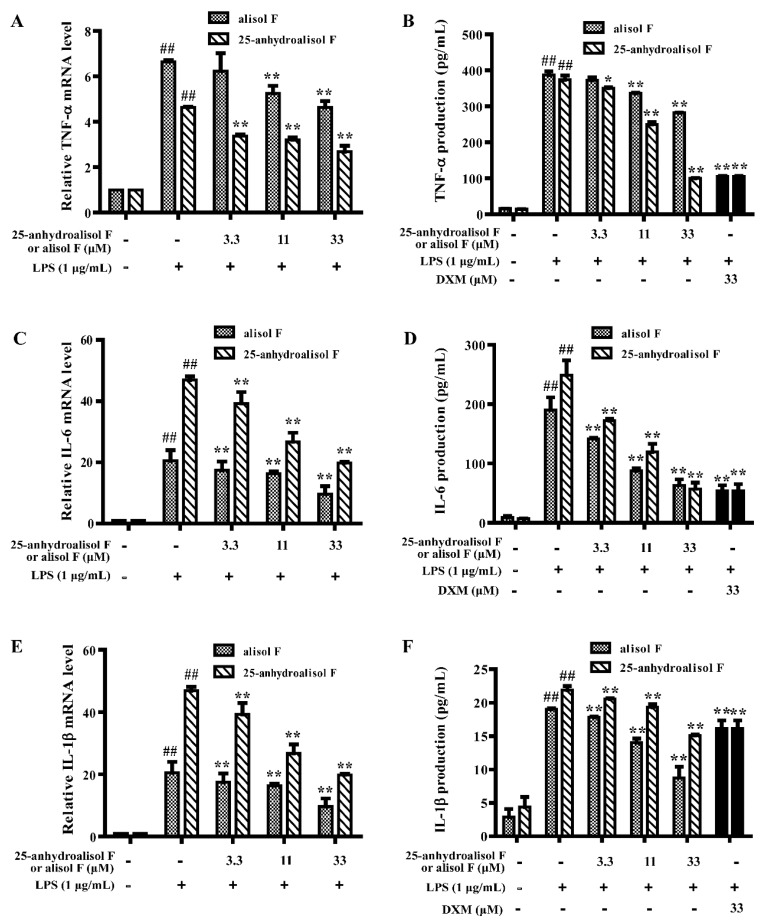
The effects of alisol F and 25-anhydroalisol F on LPS-induced expression and production of TNF-α, IL-6 and IL-1β in RAW 264.7 cells. The cells were pretreated with the different concentrations of alisol F (0, 3.3, 11 and 33 μM) and 25-anhydroalisol F (0, 3.3, 11 and 33 μM) for 2 h and then stimulated with or without LPS (1 μg/mL) for 4 h. The expression of TNF-α (**A**); IL-6 (**C**); and IL-1β (**E**) were determined by quantitative real-time PCR. The culture media were collected after 24 h LPS treatment, the production of TNF-α (**B**); IL-6 (**D**); and IL-1β (**F**) were measured using the ELISA kits. The dexamethasone (DXM) was used as the positive control. The data represent the means ± S.E. of three independent experiments. (^##^ *p* < 0.001 vs. control group; * *p* < 0.05; ** *p* < 0.001 vs. LPS group).

More closely, TNF-α, IL-6, and IL-1β played important effects on inflammation [23]. We thereby determined the levels of these pro-inflammatory cytokines. As shown in Figure 3, the secretion of TNF-α, IL-6, and IL-1β and their mRNA levels were obviously inhibited by two compounds in a dose-dependent manner in LPS-stimulated RAW 264.7 cells. All of these results further proved that alisol F and 25-anhydroalisol F had anti-inflammatory effects in vitro.

### 2.3. MAPKs, NF-κB and STAT3 Pathways Involved in Mediating the Effects of Alisol F and 25-Anhydroalisol F on Reducing Inflammation in LPS-Stimulated RAW 264.7 Cells

**Figure 4:**
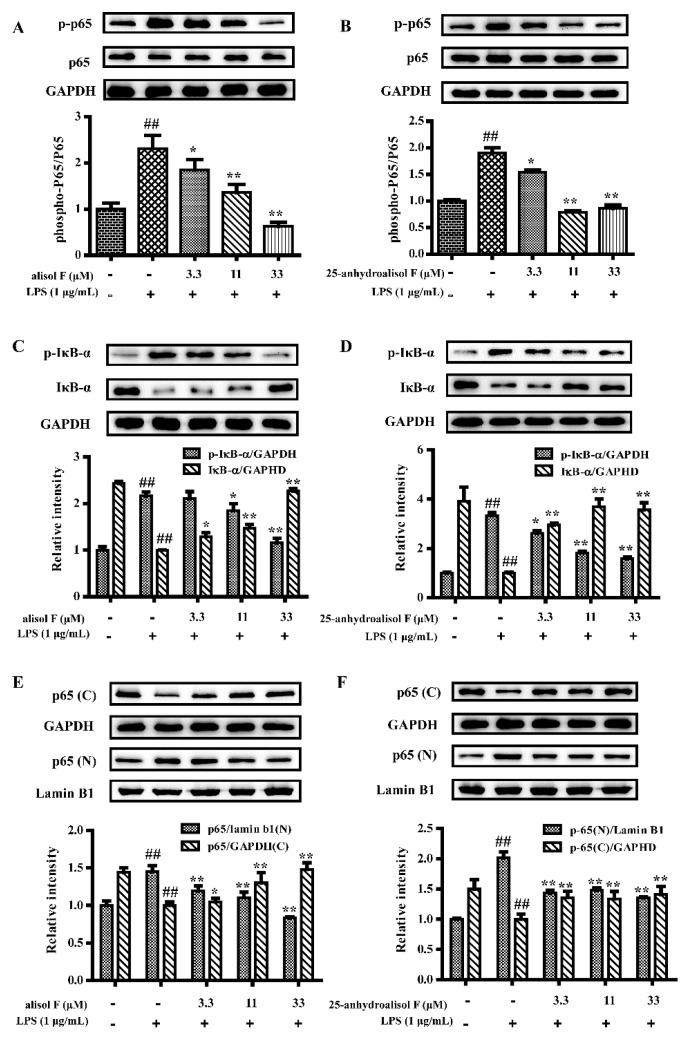
The effect of alisol F and 25-anhydroalisol F on LPS-induced activation of the NF-κB pathway in RAW264.7 cells. Cells were pretreated with the various concentrations of alisol F (0, 3.3, 11 and 33 μM) and 25-anhydroalisol F (0, 3.3, 11 and 33 μM) for 2 h and then stimulated with or without LPS (1 μg/mL) for 30 min. The expression of phospho-p65, p65, phospho-IκB-α and IκB-α were analyzed by Western blot (**A**–**D**); Cytosolic (C) and nuclear (N) protein were extracted to determine the expression of p65 by Western blot (**E**,**F**). The data represent the means ± S.E. of three independent experiments. (^##^ *p* < 0.001 vs. control group; * *p* < 0.05; ** *p* < 0.001 vs. LPS group).

**Figure 5:**
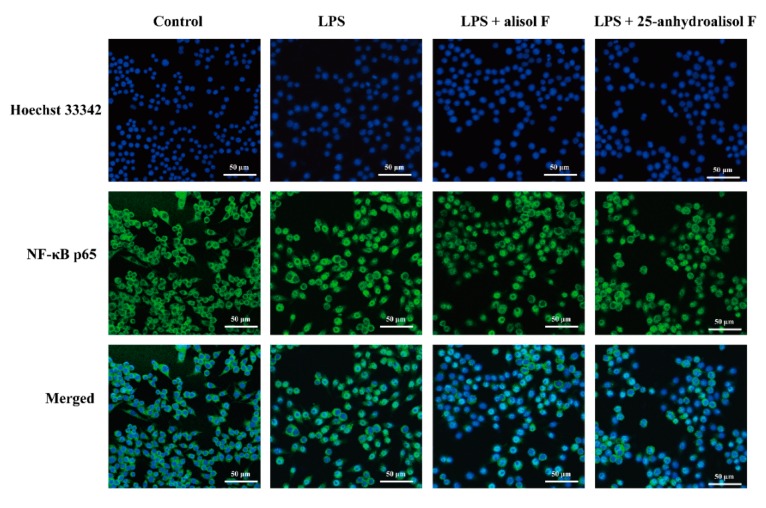
The effect of alisol F and 25-anhydroalisol F on the translocation of the NF-κB p65 subunit into the nucleus of LPS-simulated RAW 264.7 cells. Cells were pretreated with the alisol F (33 μM) and 25-anhydroalisol F (33 μM) for 2 h, and then stimulated with or without LPS (1 μg/mL) for 30 min. The translocation of NF-κB p65 was determined by immunocytochemistry. Hoechst 33342 was used to stain the nucleus of cells (blue). The cytoplasm was marked by the anti-NF-κB p65 antibody followed by fluorescein isothiocyanate (FITC)-conjugated anti-rabbit lgG (green).

In view of the above observations, the current study further investigated the molecular mechanisms of alisol F and 25-anhydroalisol F against LPS-induced inflammatory reaction in RAW 264.7 cells. As we known, NF-κB is an important transcription factor to control a variety of inflammatory cytokines such as TNF-α, IL-6, IL-1β, iNOS, and COX-2 [24,25]. To this end, we further investigated their roles in the activity of NF-κB. Western blots were initially performed to measure the phosphorylation of NF-κB p65 subunit. The results revealed that LPS (1 μg/mL) stimulated the phosphorylation of p65, which was in turn attenuated by the addition of alisol F and 25-anhydroalisol F (Figure 4A,B). As the natural inhibitor of NF-κB, the phosphorylation of IκB-α was also upregulated by the induced LPS, which resulted in the attenuation of total IκB-α in RAW 264.7 cells (Figure 4C,D). Interestingly, alisol F and 25-anhydroalisol F inhibited the phosphorylation of IκB-α, as well as increasing the protein level of IκB-α in LPS-treated cells (Figure 4C,D). Once IκB-α is phosphorylated and degraded, NF-κB p65 subunit will be released and translocated to nucleus [24]. To make it clear, we separated the cytosol and nucleus of cells and determined the protein levels of p65. Our data demonstrated that LPS increased the translocation of p65 to nucleus, which was reduced by the treatment of alisol F and 25-anhydroalisol F (Figure 4E,F). More directly, we double-stained the NF-κB p65 subunit and nucleus. The results revealed that p65 tended to translocate from the cytosol to the nucleus in LPS-stimulated cells, but the event was inhibited after alisol F and 25-anhydroalisol F treatment (Figure 5). Based on these findings, it was concluded that alisol F and 25-anhydroalisol F had the ability to attenuate the translocation of NF-κB p65 from the cytosol to the nucleus in LPS-stimulated RAW 264.7 cells.

**Figure 6:**
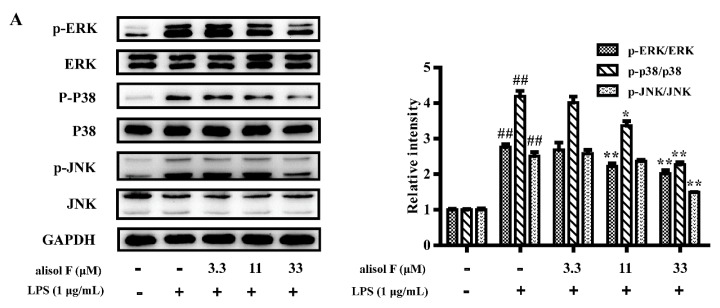

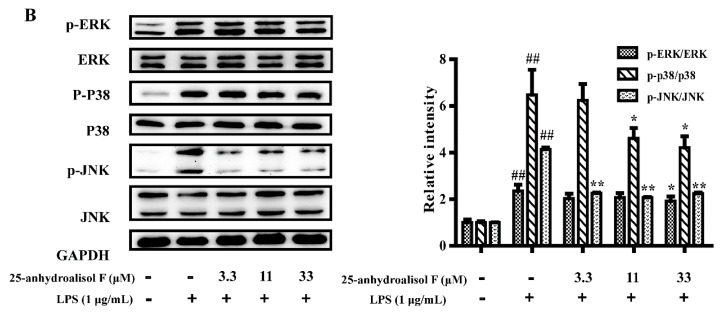
The effect of alisol F and 25-anhydroalisol F on LPS-induced activation of the MAPK signaling pathway. RAW 264.7 cells were pretreated with the different concentrations (0, 3.3, 11 and 33 μM) of alisol F (**A**) and 25-anhydroalisol F (**B**) for 2 h, then stimulated with or without LPS (1 μg/mL) for 30 min. The expressions of phospho-ERK1/2, ERK1/2, phospho-p38, p38, phospho-JNK, and JNK proteins were analyzed by Western blot. The data represent the means ± S.E. of three independent experiments. (^##^ *p* < 0.001 vs. control group; * *p* < 0.05; ** *p* < 0.001 vs. LPS group).

**Figure 7:**
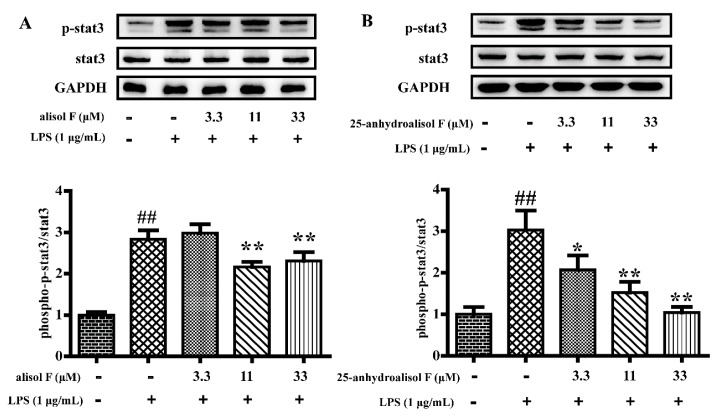
The effect of alisol F and 25-anhydroalisol F on LPS-induced activation of stat3 in RAW264.7 cells. Cells were pretreated with the different concentrations (0, 3.3, 11 and 33 μM) and of alisol F (**A**) and 25-anhydroalisol F (**B**) for 2 h and then stimulated with or without LPS (1 μg/mL) for 6 h. The expression of phospho-stat3 and stat3 proteins were analyzed by Western blot. The data represent the means ± S.E. of three independent experiments. (^##^ *p* < 0.001 vs. control group; * *p* < 0.05, ** *p* < 0.001 vs. LPS group).

In light of the roles of alisol F and 25-anhydroalisol F in NF-κB, the questions were easily raised whether the upstream signaling pathways were also regulated by the two compounds. MAPKs, including the extracellular signal-regulated kinases 1/2 (ERK1/2), p38, and c-Jun amino (N)-terminal kinases1/2 (JNK) were reported to be the upstream molecules of NF-κB [26]. To this purpose, we determined the phosphorylation of ERK1/2, p38 and JNK in LPS-treated RAW 264.7 cells. The results demonstrated that LPS activated the kinases, and alisol F and 25-anhydroalisol F clearly decreased activation of the kinases (Figure 6). Apart from MAPKs, STAT3 was also reported to be responsible for inducing the activation of NF-κB [27,28]. Similarly, we further found that alisol F and 25-anhydroalisol F attenuated the effects of LPS on stimulating the activation of STAT3 in RAW 264.7 cells (Figure 7). These results clearly demonstrated that both of compounds inhibited activation of MAPKs and STAT3 to mediate the production of pro-inflammatory cytokines.

### 2.4. Effects of Alisol F on LPS/d-gal-Induced Acute Liver Injury in Mice

**Figure 8:**
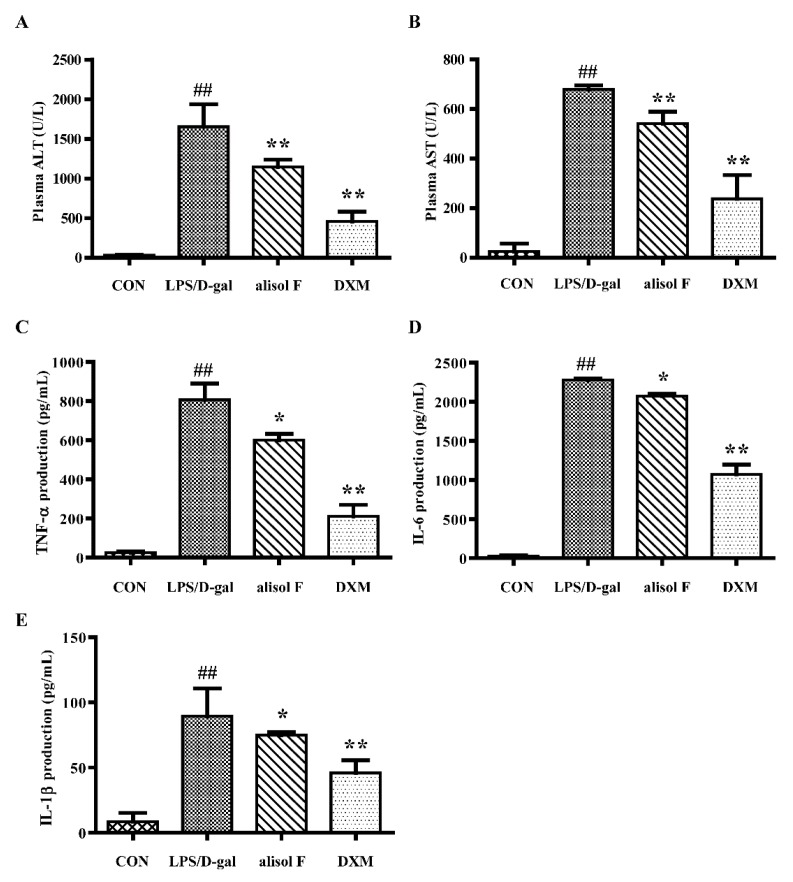
Alisol F alleviated LPS/d-gal-induced hepatotoxicity. C57BL/6 mice were pretreated with alisol F (20 mg/kg), DXM (5 mg/kg) or vehicle for three days. After 30 min of the last treatment, mice exposed to LPS/d-gal (LPS 40 μg/kg, d-gal 700 mg/kg) except for control group. Serum were harvested for measured the production of alanine aminotransferase (ALT) and aspartate aminotransferase (AST) at 5 h after LPS/d-gal exposure (**A**,**B**); Serum were harvested at 2 h after LPS/d-gal treatment for measured the production of TNF-α (**C**), IL-6 (**D**), and IL-1β (**E**) by using ELISA kits. The data represent the means ± S.E. of three independent experiments. (^##^ *p* < 0.001 vs. control group; * *p* < 0.05, ** *p* < 0.001 vs. LPS/d-gal group).

**Figure 9:**
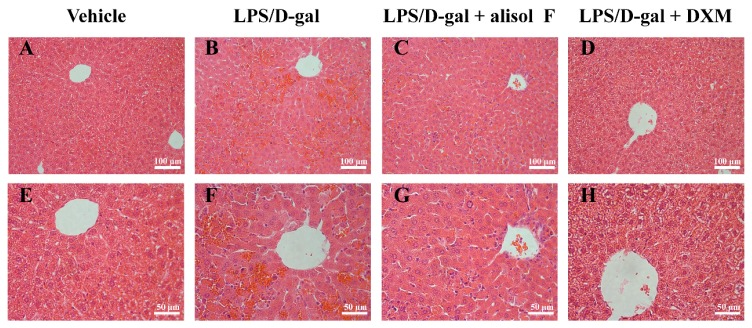
Alisol F improved histological alterations of LPS/d-gal-induced liver injury. The liver samples stained with hematoxylin-eosin (H&E) stain and acquired the image under the magnification (**A**–**D**) original magnification 200× and E-H: original magnification 400×. (**A**,**E**) mice treated with vehicles; (**B**,**F**) exposed to LPS/d-gal; (**C**,**G**) treated with Alisol F (20 mg/kg) and exposed to LPS/d-gal; (**D**,**H**) treated with DXM (5 mg/kg) and exposed to LPS/d-gal.

**Figure 10:**
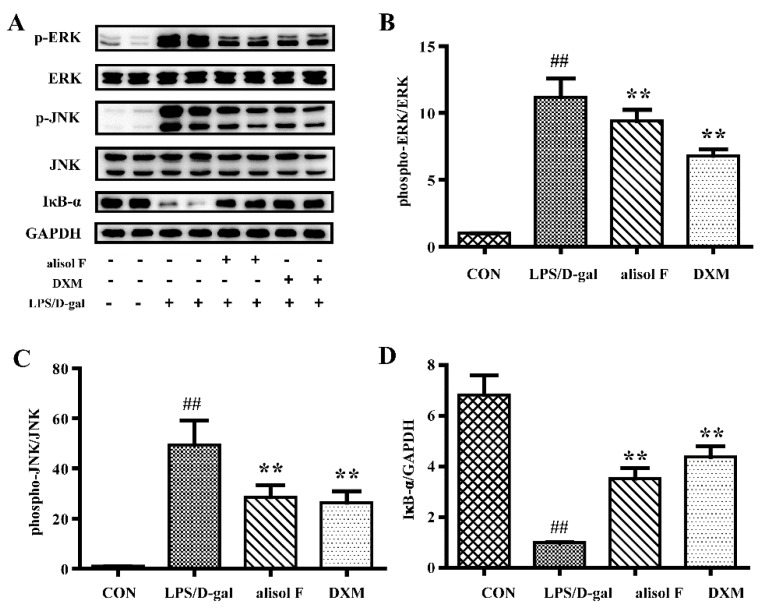
The effect of Alisol F on LPS/d-gal-induced activation of MAPKs and NF-κB signaling pathways in vivo. The mice were pretreated with Alisol F (20 mg/kg) and DXM (5 mg/kg) for three days, and induced with LPS/d-gal (40 μg/kg, 700 mg/kg). Liver samples were harvested at 5 h after LPS/d-gal treatment. The protein expression of phospho-ERK phospho-JNK and IκB-α were detected by Western blot. (**A**). Densitometry analysis of phospho-ERK (**B**), phospho-JNK (**C**) and IκB-α (**D**) were shown. The data represent the means ± S.E. of three independent experiments. (^##^ *p* < 0.001 vs. control group; * *p* < 0.05, ** *p* < 0.001 vs. LPS/d-gal group).

LPS/d-gal induced acute liver injury in mice has been widely used in investigating the mechanism underlying liver-protective and ant-inflammatory reagents [6]. To further verify the in vitro results and effects of alisol F on hepatic lesions, LPS/d-gal was employed to establish animal models of liver injury. Using this experimental models, we determined the roles of alisol F in inflammation in mice. As expected, LPS/d-gal stimulated the production of plasma alanine aminotransferase (ALT) and aspartate aminotransferase (AST) as well as the cytokines, including TNF-α, IL-6, and IL-1β, in mice (Figure 8). The addition of alisol F attenuated the effects of LPS/d-gal on the production of biomarkers in mice (Figure 8). As shown following hematoxylin and eosin (H and E) staining of liver sections (Figure 9), hepatocytes were damaged and significant hepatic sinus congestion, and inflammatory cell infiltration were observed at 5 h after LPS/d-gal-treatment. The hepatic sinus congestion was obviously improved after pretreatment with alisol F (Figure 9). The current results demonstrated alisol F exhibited clearly protective effects against LPS/d-gal-induced liver injury. In agreement with those in vitro observations, downregulation of ERK and JNK phosphorylations, as well as upregulation of total IκB-α were further confirmed in alisol F-treated liver tissues (Figure 10). Thus, these in vivo results clearly confirmed the in vitro data and suggested that alisol F protected the liver by inhibiting the inflammatory processes.

## 3. Materials and Methods

### 3.1. General Experimental Procedures

Alisol F and 25-anhydroalisol F were isolated from *A. orientale* by one of the authors (Q. Ma) using our previously-established method and provided with a purity of 98.0% determined by high-pressure liquid chromatography. Dulbecco’s Modified Eagle Medium (DMEM), fetal bovine serum (FBS), 0.25% trypsin, and penicillin-streptomycin-amphotericin (PSA) were purchased from Gibco BRL Co. Ltd. (Gaithersbug, MD, USA). Lipopolysaccharide (LPS) from *Escherichia coli* 055:B5, 3-[4,5-Dimethylthiazol-2-yl]-2,5-diphenyltetrazolium bromide (MTT), dexamethasone (DXM), d-galactosamine (d-gal) and Trizol reagent were obtained from Sigma Chemical Co. (St. Louis, MO, USA). The Griess reagent, the protein extraction kit and BCA protein assay kit were obtained from Beyotime Institute of Biotechnology (Beijing, China). Go Tag^®^ qPCR Master Mix and GoScript^TM^ Reverse Transcription System were purchased from Promega (Madison, WI, USA). Rabbit polyclonal antibodies against inducible nitric oxide synthase (iNOS), COX-2, p-p38 (Thr180/Tyr182), p38, p-ERK1/2 (Thr202/Try204), ERK1/2, p-SAPK/JNK (Thr183/Tyr185), SAPK/JNK, p-p65 (Ser536), p65, GAPDH, p-IκB-α (Ser32), IκB-α, p-STAT3 (Tyr705), STAT3, goat-IgG HRP, and lamin B1 were purchased from Cell Signaling Technology (Danvers, MA, USA). Mouse TNF-α, IL-1β, and IL-6 ELISA kit were from R and D systems (Abingdon, UK).

### 3.2. Cell Culture

The cell line RAW 264.7 was obtained from the cell bank of type culture collection of Chinese Academy of Sciences (Shanghai, China) and cultured in DMEM supplemented with 10% FBS, 2 mM glutamine, 100 U/mL of penicillin, and 100 μg/mL of streptomycin at 37 °C in a 5% CO_2_ atmosphere.

### 3.3. In Vivo Animal Model Experiments

The male C57BL/6 mice (eight-weeks old; 20–25 g) were purchased from Vital River Laboratories (Beijing, China). The animals were housed in a controlled environment under a standard room temperature, relative humidity and 12-h light/12-h dark cycle with free access to food and water for a week before experiment. All procedures were conducted in accordance with the care and use of medical laboratory animals (Ministry of Health, China, 1998) and all experimental protocols were approved by the Laboratory Ethics Committees of College of Life and Health Sciences of Northeastern University.

In order to detect the potential effects of alisol F in LPS/d-gal induced acute hepatic failure, Mice were randomly separated into four groups (*n* = 12 per group). Animals of alisol F treated group and the DXM treated group were administered alisol F (20 mg/kg, dissolved in 40% 1,2-propanediol) and DXM (5 mg/kg, dissolved in 40% 1,2-propanediol) once a day for three consecutive days by intraperitoneal injection. LPS/d-gal group and control group were treated with vehicle in the same way. After 30 min of the last treatment, acute hepatic failure of mice was induced by intraperitoneal injection of LPS and d-gal (LPS 40 μg/kg, d-gal 700 mg/kg, dissolved in saline). The control group were given the same volume of saline. After 1.5 h, six mice of each group were sacrificed to collect the serum for measuring the production of TNF-α, IL-6, and IL-1β. The other mice were sacrificed at 5 h after LPS/d-gal, then the serum were harvested for measuring activity of aminotransferases (ALT and AST) and liver tissues were collected for histological examination and Western blot analysis.

### 3.4. Measurement of Cell Viability

The cell viability was measured by MTT assay. The cells of log phase at 2 × 10^5^ cells/well were seeded in 96-well plates and incubated overnight. The cells were treated for 2 h with or without alisol F (0, 3.3, 11, 33 and 100 μM) and 25-anhydroalisol F (0, 3.3, 11, 33 and 100 μM) in the presence of LPS (1 μg/mL). After incubation for 24 h, 20 μL MTT (5 mg/mL) was added for 4 h at 37 °C, then the medium was removed and 150 μL DMSO was added to solubilize the formazan crystals of cells. The absorbance was measured at 490 nm using microplate reader (Synergy HT, BioTek, Winooski, VT, USA).

### 3.5. Determination of Pro-Inflammatory Cytokine Levels In Vivo

The RAW 264.7 cells seeded at 1 × 10^6^ cells/well in six-well plates and the pretreated with alisol F (0, 3.3, 11 or 33 μM), 25-anhydroalisol F (0, 3.3, 11 or 33 μM), and DXM (33 μM), respectively, for 2 h, and then stimulated with LPS (1 μg/mL) for 24 h. The supernatant were collected for detecting the levels of TNF-α, IL-6, and IL-1β via ELISA kit according to the manufacturer’s instructions (R and D Systems, Abingdon, UK).

### 3.6. RNA Isolation and Quantitative Real-Time PCR

Cells were seeded at 1 × 10^6^ cells/well in six-well plates and treated with alisol F (0, 3.3, 11 or 33 μM), 25-anhydroalisol F (0, 3.3, 11, or 33 μM) and DXM (33 μM) for 2 h, then stimulated with LPS (1 μg/mL) for 4 h. Total RNA was extracted with Trizol reagent and then converted to cDNA with reverse transcription system according to the manufacturer’s protocols. Expression of the certain genes were quantified by SYBR green chemistry with the Bio-Rad CFX Connect Real-Time system (Hercules, CA, USA). The expression level of the housekeeping gene GAPDH was used for standardization. The sequences of the following primers were used: TNF-α: 5′-AGCCCCCAGTCTGTATCCTT-3′ (forward), 5′-ACAGTCCAGGTCACTGTCCC-3′ (reverse); IL-1β: 5′-AGCCAAGCTTCCTTGTGCAAGTGT-3′ (forward), 5′-GCTCTCATCAGGACAGCCCAGGT-3′ (reverse); IL-6: 5′-TGTCTATACCACTTCACAAGTCGGAG-3′ (forward), 5′-GCACAACTCTTTTCTCATTTCCAC-3′ (reverse); iNOS: 5′-TGGAGCGAGTTGTGGATTGTC-3′ (forward), 5′-GGTCGTAATGTCCAGGAAGTAG-3′ (reverse); COX-2: 5′-CCAGCACTTCACCCATCAGT-3′ (forward) 5′-ACACCTCTCCACCAATGACC-3′ (reverse); GAPDH: 5′-TTGCGACTTCAACAGCAACTC-3′ (forward), 5′-GGTCTGGGATGGAAATTGTG-3′ (reverse).

### 3.7. Immunofluorescence Staining

The nuclear translocation of NF-κB p65 was analyzed by immunofluorescence assay. RAW 264.7 cells were cultured in a 24-well plate at a density of 4 × 10^5^ cells/well overnight. The cells were pretreated with 33 μM concentration of alisol F or 25-anhydroalisol F for 2 h and then stimulated with LPS (1 μg/mL) for 30 min, the cells were washed with PBS two times, fixed with 4% paraformaldehyde for 30 min at room temperature, permeabilized with 0.25% Triton X-100 for 15 min and blocked with 2% BSA for 1 h at room temperature, then washed three times with PBS and incubated with primary anti-NF-κB p65 antibody overnight at 4 °C, followed incubated with fluorescein isothiocyanate (FITC)-conjugated second antibody. After being washed with PBS, cells were incubated in 200 μL Hoechst solution for 10 min in the dark and photographed. Briefly, using a microscopy system captured the images of cells at an excitation wavelength of 350 for Hoechst and 494 nm for FITC, green fluorescence represents NF-κB p65, and blue fluorescence is the nuclei.

### 3.8. Western Blot Analysis

The RAW 264.7 cells were pretreated 2 h with alisol F (3.3, 11 or 33 μM) and 25-anhydroalisol F (3.3, 11 or 33 μM), then LPS (1 μg/mL) added to induce for 30 min, 6 h or 24 h. Total, cytoplasmic and nuclear protein was extracted via RIPA (Beyotime, Beijing, China), and the concentration measured by BCA protein assay (Beyotime, Beijing, China) according to the manufacturer’s protocols. The (20–70 μg) protein was separated with 10% SDS-PAGE and transferred onto PVDF (Millipore, Billerica, MA, USA) membrane, which was blocked for 1 h at room temperature with 5% non-fat milk in TBS. The membrane was incubated with primary antibody overnight, then washed three times with TBST and incubated with horseradish peroxidase conjugated secondary antibody for 1 h at room temperature, washed for three times with TBST and then developed by an enhanced chemiluminescence detection system (Millipore, Billerica, MA, USA). Band pattern was analyzed with Bio-Rad ChemiDoc^TM^ XRS + System (Bio-Rad, Hercules, CA, USA).

### 3.9 Analysis of Liver Enzymes and Histology

Hepatocyte damage was assessed by measuring serum enzyme activities of ALT and AST using corresponding detection kits according to the manufacturer’s instructions. Liver tissue was fixed in 4% paraformaldehyde and subsequently embedded in paraffin. Sections were stained with hematoxylin and eosin using a standard protocol and analyzed by light microscopy.

### 3.10. Data Analysis

The data were expressed as the mean ± S.E. of at least three independent experiments. The statistical significance of the differences between the means was determined either using Student’s t-test or one-way analysis of variance where appropriate. If the means were found to be significantly different, multiple pairwise comparisons were carried out by Tukey’s post hoc test. The *p* values < 0.05 (*) was considered significant, and *p* < 0.001 (**) as highly significant.

## 4. Conclusions

From the study, alisol F and 25-anhydroalisol F suppressed the LPS-induced production of NO, IL-6, TNF-α, and IL-1β, as well as iNOS and COX-2 via inhibition of the signaling pathways of MAPK, STAT3, and NF-κB in RAW 264.7 cells. Alisol F presented the protective effects against LPS/d-gal-induced liver injury in vivo. Our studies suggest that alisol F and 25-anhydroalisol F may serve as a potential lead for the development of anti-inflammatory agents for acute liver failure treatment.
